# Sealer: a scalable gap-closing application for finishing draft genomes

**DOI:** 10.1186/s12859-015-0663-4

**Published:** 2015-07-25

**Authors:** Daniel Paulino, René L. Warren, Benjamin P. Vandervalk, Anthony Raymond, Shaun D. Jackman, Inanç Birol

**Affiliations:** Canada’s Michael Smith Genome Sciences Centre, British Columbia Cancer Agency, Vancouver, BC V5Z 4S6 Canada; Department of Medical Genetics, University of British Columbia, Vancouver, BC V6H 3N1 Canada

**Keywords:** Gap closing, Genome finishing, Sealer, Next-generation sequencing, Bloom filters

## Abstract

**Background:**

While next-generation sequencing technologies have made sequencing genomes faster and more affordable, deciphering the complete genome sequence of an organism remains a significant bioinformatics challenge, especially for large genomes. Low sequence coverage, repetitive elements and short read length make *de novo* genome assembly difficult, often resulting in sequence and/or fragment “gaps” – uncharacterized nucleotide (N) stretches of unknown or estimated lengths. Some of these gaps can be closed by re-processing latent information in the raw reads. Even though there are several tools for closing gaps, they do not easily scale up to processing billion base pair genomes.

**Results:**

Here we describe Sealer, a tool designed to close gaps within assembly scaffolds by navigating de Bruijn graphs represented by space-efficient Bloom filter data structures. We demonstrate how it scales to successfully close 50.8 % and 13.8 % of gaps in human (3 Gbp) and white spruce (20 Gbp) draft assemblies in under 30 and 27 h, respectively – a feat that is not possible with other leading tools with the breadth of data used in our study.

**Conclusion:**

Sealer is an automated finishing application that uses the succinct Bloom filter representation of a de Bruijn graph to close gaps in draft assemblies, including that of very large genomes. We expect Sealer to have broad utility for finishing genomes across the tree of life, from bacterial genomes to large plant genomes and beyond. Sealer is available for download at https://github.com/bcgsc/abyss/tree/sealer-release.

**Electronic supplementary material:**

The online version of this article (doi:10.1186/s12859-015-0663-4) contains supplementary material, which is available to authorized users.

## Background

*De novo* assembly using next-generation sequencing short read sequences have been successful in producing draft genome sequences [1]. However, complete and fully automated assembly of genomes remains elusive, especially for prohibitively sized genomes such as human. Problems generally reside in areas of low-coverage or highly repetitive sequences. Even in cases where the overall long-range sequence structure can be disambiguated, on shorter scales there may be ambiguous or undetermined bases, producing regions of Ns or “gaps” in assembly scaffolds. The need for gap closure is made more evident for large (in giga-base pair, Gbp, range) genomes, such as in *H. sapiens*, where there are higher occurrences of complex genomic features. In fact, as the cost of DNA sequencing decreases faster than the cost of computer hardware, more raw sequencing data will be generated while computational resources will remain mostly the same [2]. Thus it is critical to develop tools that can scale up to these large datasets while using minimal computing resources. Projects such as the 1000 Genomes Project [3], The Cancer Genome Atlas [http://cancergenome.nih.gov/], and clinical uses of whole-genome sequencing [4] highlight the trend of processing Gbp-scale datasets.

Even though these projects are about re-sequencing human genomes and transcriptomes, it was demonstrated that *de novo* assembly of the raw reads provides valuable information on structural variations [5–8]. Thus these initiatives would benefit from a gap-closing tool that can improve the quality of human draft assemblies, while having low runtime and memory usage as it would help reduce the cost of analysis [4].

Obviously, gap-closing algorithms are also valuable in *de novo* sequencing projects, with some of the contemporary studies using the concept to improve assembly contiguity [9]. When the closed gaps refine the sequence content in or near genic or regulatory regions, they provide information for the downstream annotation work, and enable biological insights.

Hence, several tools have been designed to close gapped regions with sequence reads, including BaseClear GapFiller [10] and SOAPdenovo GapCloser [11]. The former implements a method that seeks read pairs with one pair aligning within a contig and its mate partially located in a region identified as a gap. These partially aligned reads are used to close the gap through sequence overlap. With GapCloser, a stand-alone tool in the SOAPdenovo package, reads are aligned to contig positions, and a base extension algorithm is performed. Although both of these tools have been shown to successfully close gaps in Mega-bp scale datasets such as in *S. cerevisiae* (11 Mbp) and human chromosome 14 (95 Mbp) genomes, they have difficulties to process larger datasets, such as the entire *H. sapiens* genome.

To address this need, we developed Sealer, a resource-efficient gap-filling software. Sealer uses an assembly utility within the ABySS package, called Konnector [12] as its engine to close intra-scaffold gaps. We demonstrate the scalability of Sealer on the white spruce (*P. glauca*) draft genome [13], which it processes under 27 h using 40 GB RAM – resources that can be found in contemporary commodity desktop computers. We evaluate Sealer by running tests on experimental datasets, comparing runtime, memory usage, and gap-closing success rate against state-of-the-art gap-filling applications. We expect Sealer to find a wide application for finishing small and large genomes alike.

## Implementation

### Algorithm Overview

Sealer performs three sequential functions (Additional file 1: Figure S1). First, regions with Ns are identified from an input scaffold file, and nucleotides flanking each gap are extracted. Then, flanking sequence pairs are used as input to Konnector along with a set of reads with a high level of coverage redundancy. Typically, the reads represent the original dataset from which the draft assembly is generated, or may be reads from further whole genome shotgun (WGS) sequencing data generated from the same sample. The Konnector utility is run with a range of *k-mer* lengths to connect the flanking sequences. Finally, successfully connected sequences are inserted into the gaps of the original scaffolds, and a new gap-filled scaffold file is generated. Sealer ignores size discrepancies between gaps and newly introduced sequences, since gap sizes are often estimated from fragment library distributions and assemblers do not generally provide confidence intervals for every region of Ns. Despite this, large expansions of the assembly are unlikely due to decreasing gap-closing yield of Konnector as the gap size increases [12]. Below are further details on these three steps.

- **Step 1: Extracting sequences flanking gaps.** Sealer identifies regions of Ns in an input assembly. It then searches for flanking sequences that do not contain Ns, and are at least *-L* nucleotides long (default = 100). The start position and scaffold ID of each gap are recorded for downstream processing.
- **Step 2: Konnector assembly.** The underlying engine for Sealer is Konnector, a tool to generate pseudo long reads from paired-end sequencing data by filling the unknown sequence between read pairs using the redundancy in sequence coverage. Given a *k-mer* length and a read set, Konnector builds a Bloom filters to represent all *k-mers* in the reads, and retains *k-mers* that are observed with a certain threshold multiplicity or higher (default = 2). It uses this data structure as an implicit de Bruijn graph [14] to perform a depth-limited, bidirectional, breadth-first graph search for a path that connects the flanking reads. In Sealer, gap-flanking sequence pairs are used in lieu of read pairs.

Sealer invokes Konnector with a range of *k-mer* lengths. The advantage of this “*k* sweep” strategy is, gaps with low coverage have an increased chance of being closed by shorter *k-mer* lengths, while gaps with highly repetitive sequences are more likely to be closed by larger *k-mer* values. At each *k-mer* instance, all possible traversals within the depth limit are identified between each flanking sequence pairs. Unique traversals, and traversals with path multiplicity less than or equal to a user-specified threshold (default = 2) are reported as successful connections. Consensus sequences are produced for multiple paths, reporting IUPAC ambiguity codes [15] for mismatched bases. Sealer records these generated sequences for subsequent insertion into a given scaffold.

To minimize peak memory usage, Sealer performs these local assembly runs serially, such that there is only one Bloom filter loaded at a given time. This implementation is beneficial for processing large genomes, such as *P. glauca* for which each Bloom filter instance requires 40GB RAM. Further, it allows a subtractive procedure, where we eliminate successfully closed sequence gaps from the input of subsequent iterations, minimizing CPU run time.

- **Step 3: Updating the draft assembly.** The scaffold IDs and gap start positions recorded during **Step 1** are used to match the new sequences to the corresponding gaps. However, the size of a gap and the length of a filled sequence may disagree, potentially shifting gap coordinates as Sealer closes them. To avoid this issue, Sealer processes gaps from 3′ to 5′, attempting to close the right-most gap first, moving left, making its way gap by gap towards the 5′ end of a scaffold. When a gap and new sequence are matched correctly, Sealer removes the N bases and the flanking nucleotides in the selected window, and replaces them by the newly assembled sequence. The flanking nucleotides are also replaced since the assembly process may have modified portions of these sequences, correcting micro-misassemblies near the end of the flanking sequence by adjusting them so that they are concordant with the underlying read set. This may also occur if there are alternative solutions to the original assembly problem, as in the case of polyploid genomes with allelic variations.

### Experimental Data

We used five datasets representing genomes of varying size (from 5 Mbp to 20 Gbp) and complexity (bacterial to conifer) to assess the scalability and performance of Sealer over a range of conditions (Table 1). All draft assemblies were generated using ABySS using optimized assembly parameters. Specifically, we downloaded *E. coli* K-12 substr. DH10B (~5Mbp) Illumina HiSeq 2000 paired-end reads from the Sequence Read Archive (SRA) (SRA: SRR959238). We used the reference genome [GenBank: NC_010473.1] from NCBI to assess the accuracy of the filled gaps. We closed gaps in this draft assembly with Sealer using seven *k-mer* lengths (k = 90 to 30 bp, decrementing by 10), and using the Konnector parameters -B 3000 -F 5000 -P 10. We obtained experimental *S. cerevisiae* S288c (12Mbp) data from the European Nucleotide Archive. The corresponding reference was downloaded from NCBI [GenBank:GCF_000146045.2]. Gaps were closed with Sealer using the same seven *k-mer* lengths as for *E. coli* and the Konnector parameters -B 3000 -F 700 -P 10. Experimental *C. elegans* (~100 Mbp) paired-end reads were obtained from the SRA. The latest version of the reference genome was acquired from WormBase [WormBase:WBcel235]. Sealer closed gaps in the draft assembly using 64 *k-mer* lengths (*k* =100 bp, and *k-mer* lengths between 97 and 35 bp) and the Konnector parameters -B 3000 -b 1200 M -F 700 -P 20. Sequence reads for *H. sapiens* (3.3 Gbp) individual NA19238 from the 1000 Genomes Project were obtained from the SRA, and the human genome reference GRCh38 obtained from Genome Reference Consortium. Gaps were closed with Sealer using 31 *k-mers* (250 – 130 bp, decrementing by 10 and 125 – 40 bp, decrementing by 5), and the parameters for Konnector were -B 1000 -F 700 -P10. *P. glauca* (white spruce) V3 draft assembly (20.8 Gbp) was downloaded from NCBI [GenBank:GCA_000411955.3). We ran Sealer on this dataset using two *k-mer* lengths (96 and 80 bp) while using the Konnector parameters -B 300 -F 700 -P 10.

**Table 1:** Sequence read datasets used

	*E. coli*	*S. cerevisiae*	*C. elegans*	*H. sapiens*	*P. glauca*
Coverage	615x	25x	89x	71x	65x
Read length (bp)	98	100	100	250	150, 300 and 500
Paired-end read count	14,572,674	1,582,417	44,675,422	4.68x10^8^	8.7 x10^9^
Genome size (bp)	4,686,137	12,495,682	100,258,171	3.3x10^9^	20.8x10^9^
Short Read Archive (SRA) accession	SRR959238	ERR156523	ERR294494	ERR309932	SRS357050
Gene source	GCF_000019425	GCF_000146045	GCA_000002985	Not used	Not used
ABySS version and assembly *k* parameter	v1.5.2	v1.5.2	v1.3.6	v1.5.2	v1.3.5
	*k* = 50	*k* = 30	*k* = 80	*k* = 150	*k* = 109

### Comparison to other gap-closing tools

Sealer was compared to two similar tools: GapFiller (v1.10) [10] and GapCloser (v1.12), the latter in the SOAPdenovo2 package [11]. Default settings were used for both tools in our tests, maximizing the number of compute threads, when needed (−t 16 for GapCloser on the human data set). Smaller datasets (<100 Mbp) were included in the assessment of Sealer to accommodate GapCloser and GapFiller, which have high memory and runtime requirements, respectively.

The two were also tested on the 3-gigabase *H. sapiens* draft assembly, but GapFiller was manually stopped after running for over 350 h (approximately 14 days) without completion or output. Neither one of the tools were used on *P. glauca*, based on their compute resource requirements on the high-coverage (71-fold) *H. sapiens* data.

### Machine specifications

All Sealer runs were benchmarked on a 12-core computer running CentOS 5.4 with two Intel Xeon X5650 CPUs @ 2.67 GHz and 48 GB RAM. All GapFiller and GapCloser runs were performed on a machine using CentOS 5.10 with 16 cores @ 2.13GHz, 125 GB RAM with the exception of the GapCloser run on the *H. sapiens* data. For that we used a machine running CentOS 5.9 with 16 cores @ 2.13 GHz and 236 GB RAM to accommodate the high memory requirement of GapCloser.

### Assessment of performance

To ensure consistent reporting of gap statistics, a script was developed that counts the number of regions with N bases in an assembly. This script was used to calculate the number of gaps before and after processing a draft assembly. A gap was defined as having one or a contiguous group of N bases. We used Exonerate (v2.4.0) [16] to analyze the accuracy of each tool. Sequence alignments were used to calculate the average sequence similarities (i.e. percentage of matching bases out of total bases in the query without penalizing ambiguity codes). Inserted sequences of closed gaps, rather than entire assembly scaffolds, were aligned to the corresponding reference (exonerate --percent 95). With Sealer, this was a straightforward process because of the comprehensive output it provides. Along with the new draft assembly and a log file describing specific results of each *k* run, Sealer also outputs a FASTA-formatted file of all the newly generated sequences (flanking sequences and new gap-filled sequence), which includes the original scaffold ID and gap position. In addition, there is an option to output a fasta file of gap-flanking nucleotide sequence pairs (Sealer parameter *--print-flanks*). In contrast, neither GapCloser nor GapFiller output a file of newly inserted sequences. Therefore, we had to develop a pipeline to identify and extract these novel bases for assessment and benchmarking the performance between all three tools. The pipeline begins by generating a file of all gap-flanking sequence pairs (100 bp each) found in the original assembly. It performs an Exonerate alignment using these flanking sequence pairs and a gap-filled assembly as query and target, respectively. This returns the coordinates of flanking sequence pairs within the gap-filled assembly. Using this information, the assessment pipeline extracts the bases found between each flanking sequence pair, and aligns them to the reference genome to determine sequence similarity. This pipeline was used on all three tools to maintain consistency in our analyses. We measured the runtime of Sealer using the UNIX command ‘time’. We used the Python script Memusg [https://github.com/jhclark/memusg] to benchmark peak memory usage. In addition to these analysis scripts, gap-closed assemblies of *E. coli* were manually inspected. Using parts of the sequence assessment pipeline described above, newly generated sequences and their flanking sequences were extracted from gap-closed assemblies. These sequences were aligned to the reference using Exonerate (exonerate --model affine:local [new sequence] [reference] --percent 95 --ryo Percent Identity: %pi Percent Similarity %ps). The alignments of sequences produced by Sealer were compared with the alignments of sequences generated by the other two tools. Indels (insertions and deletions), mismatches and ambiguity codes were noted. We further analyzed the quality of the new draft assemblies using QUAST [17]. Additionally, gene annotations (sources listed in Table 1), allowed us to determine whether the gaps closed by each tool spanned genic regions.

## Results and discussion

We benchmarked Sealer, GapFiller [10] and GapCloser [11] on five datasets across a broad spectrum of genome assembly sizes (~5 Mbp to 20 Gbp). The comparators were chosen based on overall performance and resource requirements, as recently evaluated [10, 17]. These studies considered several other tools that we did not include in our comparisons. IMAGE [18] was not considered based on its prohibitively high processing time [10].

**Fig. 1:**
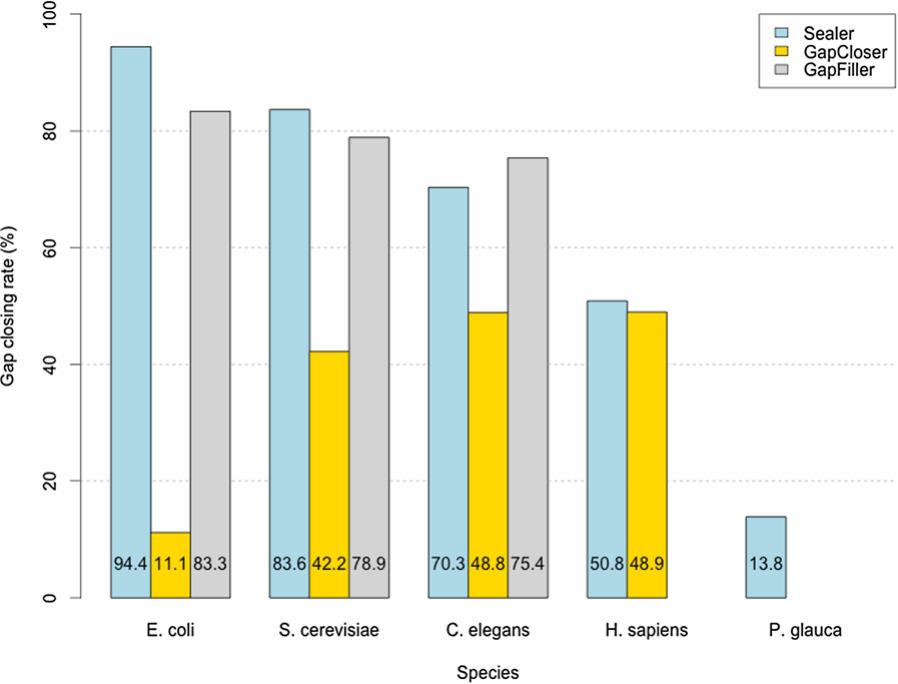
Gap-closing success rates. Results of gap closure of the tools tested on a broad-size genome data spectrum (5 M to 20 Gbp). Baseclear GapFiller could not complete its run on *H. sapiens*. Both GapFiller and GapCloser were not attempted on the *P. glauca*, due to their high resources requirements

Gap-closing capability was similar between GapFiller and Sealer (Fig. 1), with Sealer outperforming the former in two out of three small draft genomes (<1 Gbp). GapCloser never achieved a full gap closing success rate above 50 % in any of the datasets tested. We note that GapFiller and GapCloser both have the ability to resolve some of the unambiguous bases within a gap, even when the gap is not completely closed. In contrast, Sealer produces an all-or-none gap closing output. A summary of the comparison results are presented in Table 2.

**Table 2:** Gap-closing performance of Sealer, SOAPdenovo GapCloser and GapFiller on five draft genome assemblies ranging from ~5 Mbp to 20 Gbp

Draft genome species	Total gaps	Software	Gaps completely closed	% Success	Wall clock time (hh:mm)	Memory (GB)
*E. coli*	18	Sealer	17	94.4	00:20	0.5
		GapCloser	2	11.1	00:05	25.7
		GapFiller	15	83.3	00:43	0.4
*S. cerevisiae*	213	Sealer	178	83.6	00:02	0.5
		GapCloser	90	42.3	00:02	3.8
		GapFiller	168	78.9	00:20	0.7
*C. elegans*	4,223	Sealer	2,968	70.3	09:54	1.4
		GapCloser	2,062	48.8	01:49	101.0
		GapFiller	3,186	75.4	37:42	0.7
*H. sapiens*	237,406	Sealer	120,676	50.8	29:19	22.2
		GapCloser	116,297	48.9	83:15	178.1
		GapFiller	Incomplete. Terminated after 353 h.			
*P. glauca*	2,894,274	Sealer	399,476	13.8	26:12	45.3
		GapCloser	Not attempted			
		GapFiller				

We took advantage of the small size of *E. coli* to manually inspect every sequence produced by Sealer, GapCloser and GapFiller. We aligned these sequences to the reference genome to determine percent similarities. As shown in Additional file 2: Table S1, Sealer closed 17 of the 18 gaps while GapCloser and GapFiller fully closed 2 and 15 gaps, respectively. Out of the 17 new sequences output by Sealer, 15 had 100 % similarity to the reference genome. GapCloser also had 100 % similarity, but only from its two fully closed sequences. GapFiller performed similarly to Sealer, obtaining 100 % similarity for all but 2 gaps. The gaps commonly closed by all 3 tools (n = 2) comprised the same base sequence. Furthermore, the sequences of commonly closed gaps between Sealer and GapFiller were the same, with the exception of two gaps. Three sequences produced by Sealer contained ambiguity codes, consistent with the ability of Konnector to report alternate bases when multiple assembly paths are possible.

For *S. cerevisiae*, of the 52 commonly closed gaps (out of 213), percent similarities to the reference genome are 99.94 %, 99.96 % and 99.95 % for Sealer, GapCloser and GapFiller respectively. Sealer successfully closed 178 of the 213 gaps in this dataset, outperforming the other tools.

**Fig. 2:**
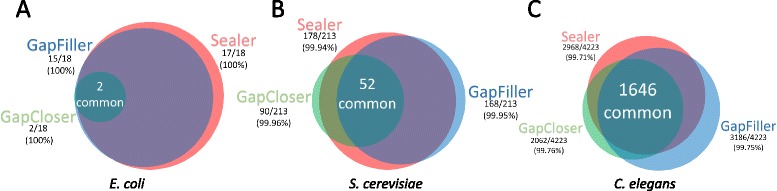
Identity of closed gaps by Sealer and two leading gap-filling applications. Venn diagrams depict the overlap of gaps closed between each tool for the **a**) *E. coli*, **b**) *S. cerevisiae* and **c**) *C. elegans* datasets. The sizes of individual circles represent the number of gaps closed relative to the other tools. Overlapping closed gaps were approximated using the assessment pipeline described in Section 2.5 and depicted using the online VennDiagram.tk tool [http://www.cmbi.ru.nl/~timhulse/venn/]

For *C. elegans*, 1646 gaps were detected as commonly closed. Sequence similarities were reported as 99.71 %, 99.76 % and 99.75 % for Sealer, GapCloser and GapFiller respectively. These findings (summarized in Table 2 and Fig. 2) show that the accuracy of the gaps closed by the three tools are comparable, while the success rate of Sealer is the highest in all but one experiment.

We note that the peak memory usage of GapCloser is up to one hundred times higher compared to Sealer (Table 2 and Additional file 3: Figure S2, eg. 101 GB vs 1.35 GB for *C. elegans*), and memory requirements are similar between Sealer and GapFiller. For smaller genomes, GapCloser had the fastest run times, but consistently closed less gaps compared to the other two tools. GapFiller had the slowest run time for all the experiments. When closing gaps in the *H. sapiens* draft assembly, GapFiller was left running for over 353 h (~2 weeks) before we manually killed the process. GapCloser was able to complete this dataset in three and a half days using 178.1 GB RAM, while GapFiller was still processing 19 % of the paired-end reads at the time of its termination, with no partial assembly output. Sealer closed 120,676 of the 237,406 human draft assembly gaps, a success rate of 50.8 %, in less than 30 h (~1 day, 5 h) compute time. Compared to GapFiller, Sealer used ~8 times less memory to close marginally more gaps. Likewise, Sealer processed the colossal white spruce 20 Gbp draft assembly in 26.2 h using Bloom filters for two values of *k*, and achieved a gap-closing success rate of 13.8 %, closing 399,476 of the 2,894,274 gaps in the NCBI V3 draft assembly.

The results reported by QUAST (Additional file 4: Table S2) further supports the ability of Sealer to produce quality draft assemblies. With the exception of the *C. elegans* assemblies, the resulting Sealer gap-filled assemblies are contiguous, even when factoring mis-assemblies, as the NGA50 length metric suggests. This indicates that the mis-assemblies in the Sealer *E. coli* and *S. cerevisiae* assemblies tend to be on shorter scaffolds. In all genomes under study, Sealer was marginally superior in its ability to close gaps located within genes compared to the other tools. Average additional number of complete genes recovered by Sealer compared to other applications for *E. coli, S. cerevisiae* and *C. elegans* is 8.5 +/− 6.4, 10.5 +/− 12.0 and 20.0 +/− 1.4 in that order. We speculate that, when measuring number of Ns per 100 kbp, QUAST is counting ambiguity codes as well, since no N bases exist in the contig files submitted to the analysis tool.

Leading gap-filling applications, namely GapFiller and SOAPdenovo’s GapCloser resolve gaps by short read sequence alignments. Current implementations of this paradigm have similar gap-filling efficiencies on small bacterial genomes [10], which is comparable to that of Sealer. They however, have difficulty scaling to larger genomes such as the whole human genome and sizes beyond. This will become increasingly more important as the data throughput from sequencing instruments continues to swell, and researchers undertake more *de novo* sequencing projects of large genomes. For such projects, even though it is possible to process assembly scaffolds in smaller batches on a compute farm, doing so would result in an overhead that is often impractical and requires specialized hardware and bioinformatics know-how. While our manuscript was in review we became aware of a promising, but not yet scalable, graph-based gap filling algorithm [19], which was reported at the time to close 28 % more gaps in bacterial genome assemblies when compared to GapFiller and GapCloser.

Sealer uses a light-weight Bloom filter de Bruijn graph assembler as its core assembly algorithm. This gives Sealer a few advantages over alternative tools. 1) The entire 1.35 Tbp *P. glauca* read set [13] easily fits on a 48 GB RAM computer, a system that is now accessible to most labs. 2) The *de novo* assembly method Sealer uses has the advantage of generating multiple paths through a *k-mer* graph, which could be used to effectively capture allelic differences and sequence variants, both key features to genetics and cancer studies. In contrast, GapCloser and GapFiller use coverage information and a threshold score to determine which consensus base is used in the case of a discrepancy, effectively stripping that information. 3) The fast bi-directional de Bruijn graph assembly strategy, specifically allows one to exhaustively assemble *k-mers* across gaps using a comprehensive range of *k* values. We applied this strategy to the *C. elegans* dataset, testing 64 different *k* values (thus iteratively building 64 Bloom filters). Although this impacted its run time, Sealer was still four times faster than GapFiller while closing above 70 % of the gaps, a yield comparable to that obtained by the latter. Further, because the Sealer run time is low and its memory footprint is relatively small, one could envision building Bloom filters with additional data from the same sample, or even third-party data from same-species, to produce a mosaic assembly in a manner similar to the human genome reference.

When running individual Sealer runs at unique values of *k* on 250 bp human experimental WGS read data, we find that *k* = 80 is more successful at closing gaps. When considering the entire *k* spectrum, being more permissive on the maximum number of assembly paths (from –P 2 to –P 10) increases gap closure by 9.8 % overall (Additional file 5: Figure S3A). Generally speaking, *k-*mers varying in size from 60 to 220 bp were all suited to close gaps in the human draft assembly, and gaps of equivalent sizes tend to close in a *k*-independent manner, with a slight constriction of gap length distribution with decreasing *k* (Additional file 5: Figure S3B). This is not surprising since Konnector achieves maximum efficiency on fragments < 1 kbp [12]. On a practical note we recommend, whenever possible, exploring a wide range of *k*, typically from the read length *L* to *k* = 40, which is the practical lower limit of *k* for Konnector. For human, we ran Sealer iteratively by exploring 31 *k*-mers from 250 to 40 nucleotides, and still completed the run in less than half the time compared to the next best application (Table 2). When working with de Bruijn graphs, shorter *k*-mers yield more tangled graphs but are useful when read coverage is inadequately low. On the flip side, the use of longer *k*-mers help disambiguate repeats and tangles in the graph when the coverage is sufficient, and are expected to help resolve gaps that arise due to repeats [20]. We find that, when used iteratively, both large and short *k*-mers close a similar number of gaps in the human data set (Additional file 6: Table S3). A combination of *k*-mers used in iterative cascading runs is thus warranted since, clearly, lower *k* values close more than half of the gaps that were not successful at larger values of *k*. Also, larger *k*-mer values tend to close larger gaps and overlaps. Generally, the first *k-*mer utilized closes the most gaps in iterative runs (Additional file 7: Figure S4A), which is why running Sealer first with large *k* values that can more readily resolve repeats is recommended. We profiled the repeat content in gaps [21] that were uniquely closed at a specific *k*-value during the iterative Sealer run on the human assembly. We observe that gaps with SINEs are preferentially closed at a shorter *k* while those comprised of simple repeats, LINEs and Satellites are preferentially closed with larger *k*-mers, which is intuitive. Overall, we do not find many gaps harboring low complexity regions, small RNA, and unclassified repeats, suggesting that we may have limited success in closing gaps comprising these features (Additional file 7: Figure S4B). We have randomly sampled 350 gaps that we could not close after the Sealer iterative *k* runs on the human assembly draft. We observe that 95.6 % +/− 2.0 % of those are due to repeats in the hg19 reference human genome, as indicated by the corresponding regions harboring lower case bases in the reference genome. The +/− 2.0 % interval refers to the 95 % confidence interval of this estimated rate.

GapCloser and GapFiller will resolve base ambiguities as they iterate through a subset of sequences even when they cannot fully close a gap. Sealer on the other hand provides an all-or-nothing output, not reporting partially filled gaps when the number of possible reconstructions between flanking gap sequences exceed a user-defined threshold or when assembly paths are obscured. As a result, GapCloser and GapFiller results may have fewer Ns per 100 kbp (Additional file 4: Table S2). However, we note that remaining gaps still require design and execution of finishing experiments, and arguably fewer gaps would be desirable over more but slightly shorter gaps. Also, because GapCloser and GapFiller do not report their partially closed gaps, we were not able to test their accuracy.

## Conclusions

Finishing genomes has been relevant since sequencing *H. influenzae,* the first shotgun genome assembly [22] and, more than ten years after publishing of the first human genome draft, we still do not have a complete assembly [23]. There have been few reasons for that, including difficulty in cloning, sequencing and assembling heterochromatin, as well as the lengthy, tedious nature of finishing work. But the prohibitive analysis cost is one of the main reasons why we do not completely finish genomes today. With DNA sequencing affordability on the rise [4], shotgun assembly of large genomes (>1 Gbp) is increasingly becoming routine in research laboratories, and widespread uptake in the clinic is anticipated. Consequently, obtaining better draft genomes is a common goal of all *de novo* sequencing projects. Further, *de novo* assembly and sequence finishing is also finding applications in many re-sequencing projects, especially in oncogenomics projects where comprehensive sequencing and nucleotide-level resolution informs clinical intervention [24]. Sealer is a scalable gap-filling software expected to be an indispensable addition to the genome finishing toolkit, and with broad application to on-going finishing efforts.

Whereas obtaining 100 % completion is unlikely without at least some computer-assisted manual finishing and labour-intensive PCR work, Sealer brings human genome finishing and finishing of colossal genomes such as that of the 20 Gbp white spruce one-step closer.

### Availability and requirements

**Project name:** Sealer

**Project home page:**https://github.com/bcgsc/abyss/tree/sealer-release

**Operating system(s):** UNIX

**Programming language:** C++

**Other requirements:** Boost C++ library (headers only) and Google sparsehash library

**License:** GNU GPL

**Any restrictions to use by non-academics:** license needed

## Additional files

Additional file 1: Figure S1. Sealer gap-closing pipeline. PDF file depicting the steps to gap-filling genome draft assemblies with Sealer. Additional file 2: Table S1. Table of gaps closed in the *E. coli* K-12 genome. PDF file presenting an in-depth gap sequence analysis in *E. coli*. Additional file 3: Figure S2. Compute resource required. PDF file showing benchmarking results of Sealer, SOAPdenovo GapCloser and GapFiller for closing gaps in draft genome assemblies. Additional file 4: Table S2. Table detailing a quality assessment of gap-filled assemblies. PDF file reporting a QUAST analysis of gap-closed *E. coli, S. cerevisiae* and *C. elegans* draft genome assemblies. Additional file 5: Figure S3 Individual Sealer *H. sapiens* runs at unique values of -*k* and –*P. (PDF 126 kb)*
Additional file 6: Table S3 Closing gaps in a human genome draft assembly using Sealer with different *k* value ranges. (PDF 54 kb) Additional file 7: Figure S4. Closing gaps iteratively in a cascading fashion at various values of *k* in the *E. coli*, *S. cerevisiae*, *C. elegans*, *H. sapiens* and *P. glauca* draft genomes.

## Abbreviations

ABySS: Assembly By Short Sequences
WGS: Whole Genome Shotgun
IUPAC: International Union of Pure and Applied Chemistry
RAM: Random Access Memory
Gbp: Giga base pairs
CPU: Central Processing Unit
