# Supplementary material for: Sealer: a scalable gap-closing application for finishing draft genomes
Source: BMC Bioinformatics. 2015 Jul 25;16(1):230. doi: 10.1186/s12859-015-0663-4 (PMC4515008; doi:10.1186/s12859-015-0663-4)
Supplement: Additional file 1: Figure S1. — Sealer gap-closing pipeline. PDF file depicting the steps to gap-filling genome draft assemblies with Sealer. [file 12859_2015_663_MOESM1_ESM.pdf]

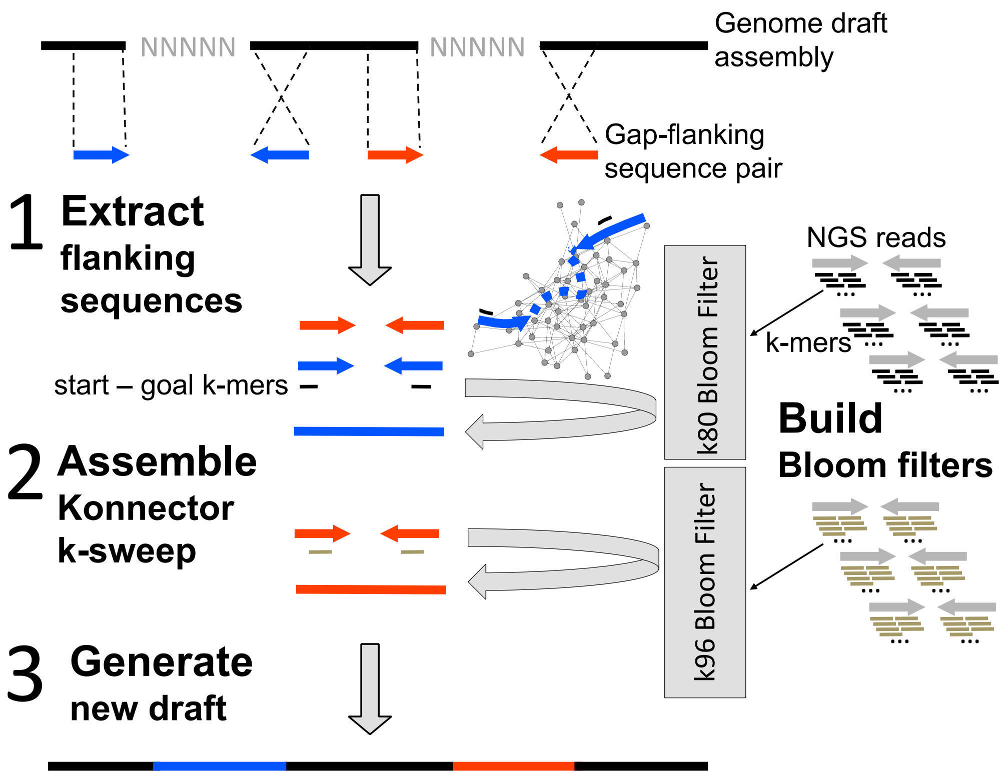

Supplemental figure S1. Steps to gap-filling genome draft assemblies with Sealer. Gaps are first detected from a draft assembly and flanking sequence pairs extracted. Next-generation sequence reads are supplied and *k*-1 overlapping *k-mers* extracted and used to build Bloom filters. Bloom filters are interrogated in turn using start and goal *k-mers* from each flanking pairs and sequences are derived by navigating a de Bruijn graph. Resulting sequences are merged back in the original draft assembly producing a new, gap-filled genome sequence.
