# Supplementary material for: Sealer: a scalable gap-closing application for finishing draft genomes
Source: BMC Bioinformatics. 2015 Jul 25;16(1):230. doi: 10.1186/s12859-015-0663-4 (PMC4515008; doi:10.1186/s12859-015-0663-4)
Supplement: Additional file 2: Table S1. — Table of gaps closed in the E. coli K-12 genome. PDF file presenting an in-depth gap sequence analysis in E. coli. [file 12859_2015_663_MOESM2_ESM.pdf]

Supplemental table S1. In-depth gap sequence analysis of the *E. coli* K12 genome.

Gap ID	Original assembly gap length (bp)	Sealer gap length (bp)	GapCloser gap length (bp)	GapFiller gap length (bp)
1469_246	50	1	-	1
1470_4783	50	-8	-8	-8
1470_5078	50	-9	-9	-9
1471_1044	50	10*	-	8
1472_386	50	-4	-	-4
1473_1283	50	7**	-	-
1476_1501	50	0	-	0
1477_12528	50	8	-	8
1478_5345	69	-11	-	-11
1479_429	50	391	-	-24[†]
1480_12947	50	-1	-	-1
1481_446	50	8[‡]	-	8[‡]
1482_9410	50	958[§]	-	-
1483_3927	50	-17	-	-17
1484_541	50	-	-	-
1485_676	50	-22	-	-22
1487_3816	50	-43	-	-43
1488_3131	50	1	-	1

A negative number indicate an overlap at the gap location. Absence of a value indicate that the particular gap was not fully closed.

**2 insertions, 1 ambiguity code*

*** 2 ambiguity codes*

† 169/176 aligned bases, 96% similarity

‡ 1 mismatch, 99.52% similarity

§ 1 ambiguity code

All results aligned to the reference genome with 100% similarity unless otherwise stated.
