# Supplementary material for: Sealer: a scalable gap-closing application for finishing draft genomes
Source: BMC Bioinformatics. 2015 Jul 25;16(1):230. doi: 10.1186/s12859-015-0663-4 (PMC4515008; doi:10.1186/s12859-015-0663-4)
Supplement: Additional file 3: Figure S2. — Compute resource required. PDF file showing benchmarking results of Sealer, SOAPdenovo GapCloser and GapFiller for closing gaps in draft genome assemblies. [file 12859_2015_663_MOESM3_ESM.pdf]

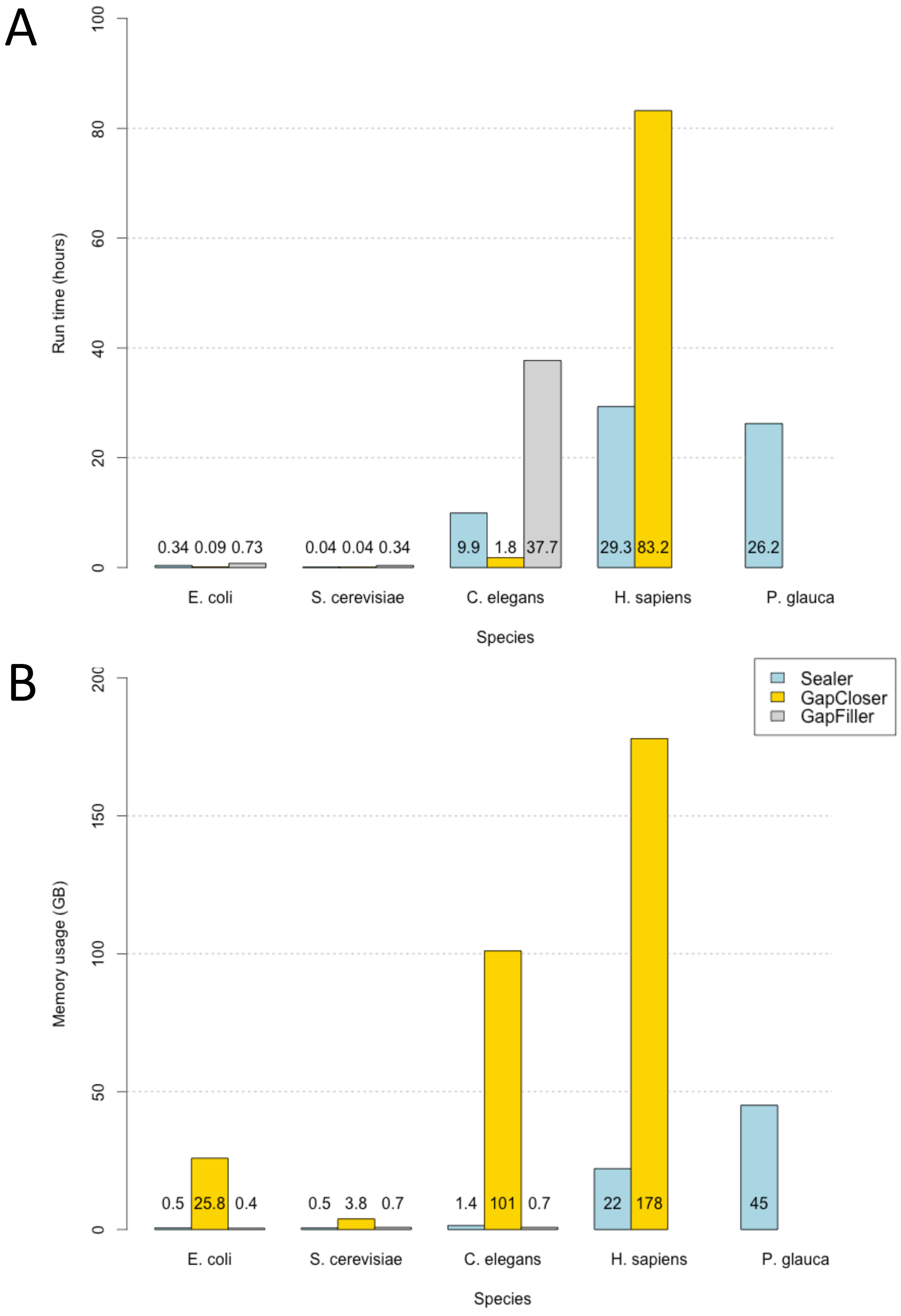

Supplemental figure S2. Compute resource required by Sealer, GapCloser and GapFiller for closing gaps in the *E.coli* K12, *S. cerevisiae*, *C. elegans*, *H. sapiens* and *P. glauca* draft genome assemblies. A) Run time B) Memory usage.
