# Supplementary material for: Sealer: a scalable gap-closing application for finishing draft genomes
Source: BMC Bioinformatics. 2015 Jul 25;16(1):230. doi: 10.1186/s12859-015-0663-4 (PMC4515008; doi:10.1186/s12859-015-0663-4)
Supplement: Additional file 4: Table S2. — Table detailing a quality assessment of gap-filled assemblies. PDF file reporting a QUAST analysis of gap-closed E. coli, S. cerevisiae and C. elegans draft genome assemblies. [file 12859_2015_663_MOESM4_ESM.pdf]

Supplemental table S2. QUAST analysis of gap-closed *E. coli, S. cerevisiae* and *C. elegans* draft genome assemblies.

	E. coli K12 (4,686,137 bp)				*S. cerevisiae* S288c (12,071,326 bp)				*C. elegans* Bristol N2 (100,272,607 bp)			
	Original assembly	Sealer	GapCloser	GapFiller	Original assembly	Sealer	GapCloser	GapFiller	Original assembly	Sealer	GapCloser	GapFiller
Number of sequences*	535	**526**	534	527	1750	**1682**	1737	1687	5846	4701	4970	**4179**
Largest sequence (bp)	66338	66338	66338	66338	59854	59854	59854	59854	384642	1349218	1349228	762554
N50 (bp)	14251	**14601**	14251	**14601**	10397	**10724**	10429	**1072**4	39508	52267	48508	**59358**
NG50 (bp)	13120	**13190**	13120	13150	9497	**9963**	9626	9934	41165	55074	51773	**59732**
Mis-assemblies	**2**	3	**2**	**2**	**14**	15	**14**	**14**	**231**	440	370	362
N's per 100 kbp	0.00	0.09	0.00	0.00	1.25	1.45	0.00	0.00	1.54	5.58	0.00	0.00
Mis-match per 100 kbp[1]	**2.01**	2.67	**2.01**	3.06	81.02	82.19	82.53	**80.60**	**15.53**	22.24	22.81	20.63
Complete genes	3765	**3787**	3771	3779	5089	**5149**	5103	5122	47405	**47743**	47645	46269

* All statistics are based on sequences of size >= 500

1 Sealer introduces IUPAC ambiguity codes when a base is uncertain, which is assessed as a mismatch by QUAST
