# Supplementary material for: Sealer: a scalable gap-closing application for finishing draft genomes
Source: BMC Bioinformatics. 2015 Jul 25;16(1):230. doi: 10.1186/s12859-015-0663-4 (PMC4515008; doi:10.1186/s12859-015-0663-4)
Supplement: Additional file 5: Figure S3 — Individual Sealer H. sapiens runs at unique values of -k and –P. (PDF 126 kb) [file 12859_2015_663_MOESM5_ESM.pdf]

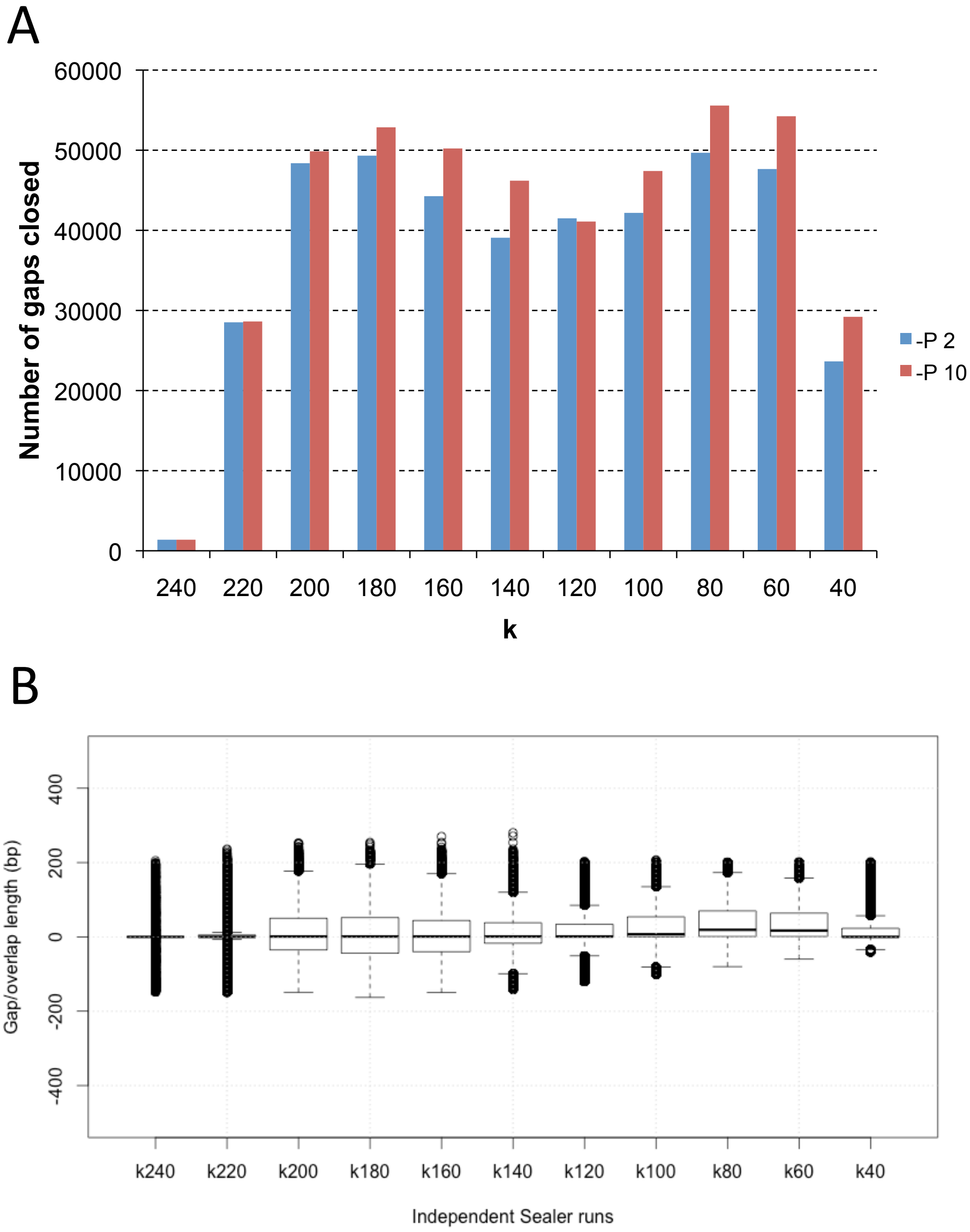

Supplemental figure S3. Individual Sealer *H. sapiens* runs at unique values of -*k* and -*P*. A) Gap closing yield as a function of *k* and varying with the maximum number of path allowed. B) gap size distribution for individual Sealer runs at unique values of *k*.
