# Supplementary material for: Sealer: a scalable gap-closing application for finishing draft genomes
Source: BMC Bioinformatics. 2015 Jul 25;16(1):230. doi: 10.1186/s12859-015-0663-4 (PMC4515008; doi:10.1186/s12859-015-0663-4)
Supplement: Additional file 6: Table S3 — Closing gaps in a human genome draft assembly using Sealer with different k value ranges. (PDF 54 kb) [file 12859_2015_663_MOESM6_ESM.pdf]

Supplemental table S3. Closing gaps in a human genome draft assembly using Sealer with different *k* value ranges.

	Run 1 (k=250 – 120, step 10)	Run 2 (k=125 – 40, step 5)	Run 1 + Run 2 combined
Number of gaps closed	58,432	62,244	120,676
% closed	24.6	26.2	50.8
Average gap size (± S.D. bp)	5.55 ± 72.73	55.72 ± 73.77	31.58 ± 77.40
Longest gap closed (bp)	195	403	403
Longest overlap closed (bp)	149	125	149
