# Supplementary material for: Sealer: a scalable gap-closing application for finishing draft genomes
Source: BMC Bioinformatics. 2015 Jul 25;16(1):230. doi: 10.1186/s12859-015-0663-4 (PMC4515008; doi:10.1186/s12859-015-0663-4)
Supplement: Additional file 7: Figure S4. — Closing gaps iteratively in a cascading fashion at various values of k in the E. coli, S. cerevisiae, C. elegans, H. sapiens and P. glauca draft genomes. [file 12859_2015_663_MOESM7_ESM.pdf]

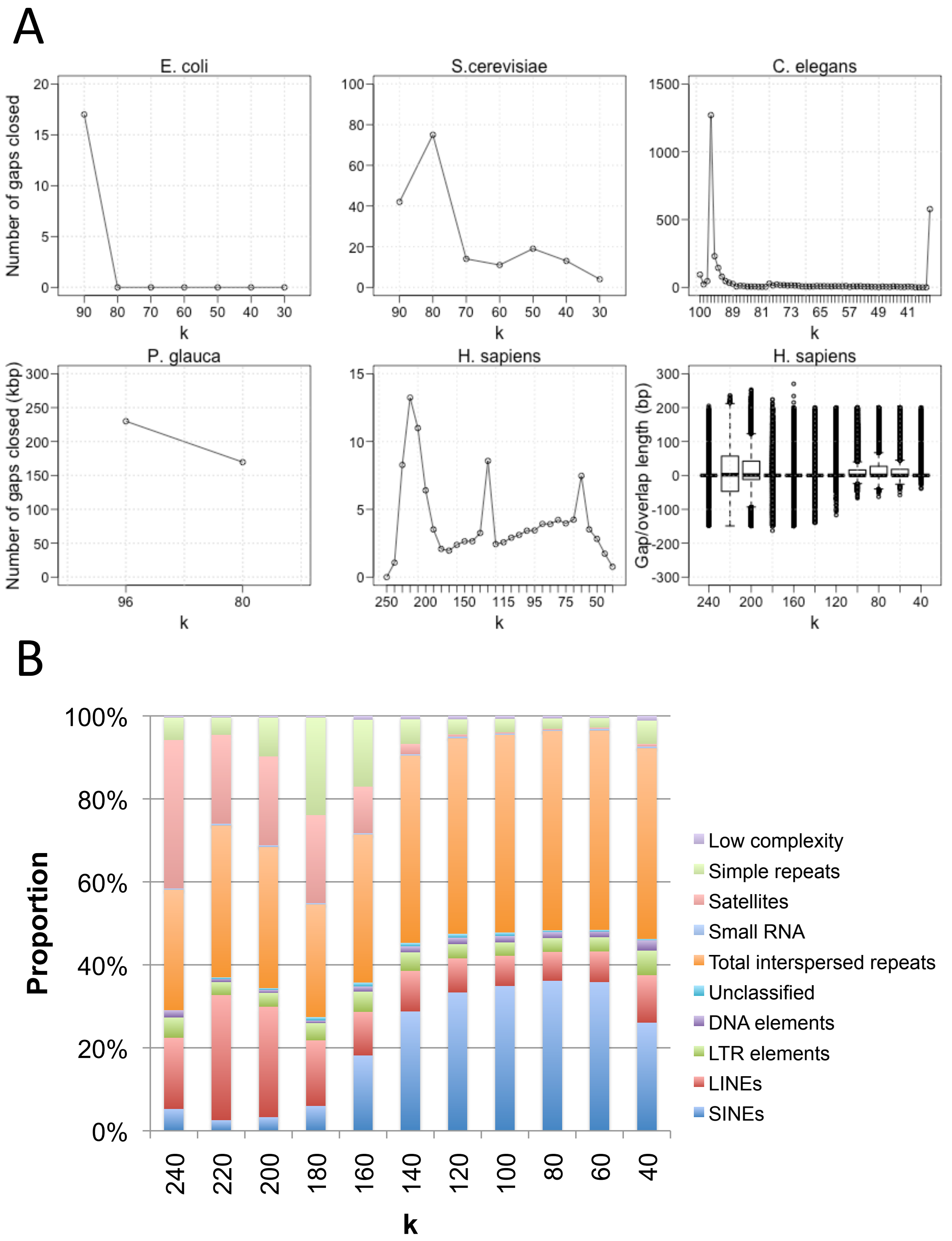

Supplemental figure S4. Closing gaps iteratively in a cascading fashion at various values of *k* in the *E. coli*, *S. cerevisiae*, *C. elegans*, *H. sapiens* and *P. glauca* draft genomes. A) Gaps were closed iteratively using a wide range of *k*-mer values, as described in methods. As a rule of thumb, we start with the largest *k*-value first in order to more readily resolve repeats, except for *C. elegans* where *k*=35 ran before *k*=96 (peak at 1270). When running iteratively with a range of *k*-value, the

first *k* used will typically close the most gaps, except when this value approaches the read length. Bottom right panel: Gap size distribution for gaps closed by Sealer in the human data set at *k*240-40. **B)** Repeat content analysis in those corresponding *H. sapiens* gap sequences. We ran RepeatMasker (Smit et al., 2013-2015) on gaps that were exclusively closed at the specific *k* values presented in A), bottom right panel.

Supplemental References

Smit, AFA, Hubley, R & Green, P. RepeatMasker Open-4.0.
2013-2015. http://www.repeatmasker.org.
